# Obesity and risk for its comorbidities diabetes, hypertension, and dyslipidemia in Japanese individuals aged 65 years

**DOI:** 10.1038/s41598-023-29276-7

**Published:** 2023-02-09

**Authors:** Tomoko Yamada, Maki Kimura-Koyanagi, Kazuhiko Sakaguchi, Wataru Ogawa, Yoshikazu Tamori

**Affiliations:** 1 Department of Nutrition, Kobe University Hospital, 7-5-2 Kusunoki-cho, Chuo-ku, Kobe, 650-0017 Japan; 2 Division of Diabetes and Endocrinology, Department of Internal Medicine, Kobe University Graduate School of Medicine, 7-5-1 Kusunoki-cho, Chuo-ku, Kobe, 650-0017 Japan; 3 Integrated Clinical Education Center, Kobe University Hospital, 7-5-2 Kusunoki-cho, Chuo-ku, Kobe, 650-0017 Japan; 4 Division of General Internal Medicine, Department of Internal Medicine, Kobe University Graduate School of Medicine, 7-5-1 Kusunoki-cho, Chuo-ku, Kobe, 650-0017 Japan; 5 Division of Creative Health Promotion, Department of Social/Community Medicine and Health Science, Kobe University Graduate School of Medicine, 7-5-1 Kusunoki-cho, Chuo-ku, Kobe, 650-0017 Japan

**Keywords:** Health care, Weight management, Risk factors

## Abstract

Diabetes, hypertension, and dyslipidemia are obesity-related comorbidities that contribute to the development of cardiovascular disease, one of the leading causes of death. In addition to obesity, the underweight condition is a concern because it can give rise to sarcopenia, particularly after the age of 65 years. We examined the risk for diabetes, hypertension, and dyslipidemia due to obesity in individuals of this age. We retrospectively investigated the relation between obesity and its three major comorbidities in 10,852 individuals aged 65 years who underwent health checkups implemented by Kobe City between April 2017 and March 2021. The prevalence of diabetes, hypertension, and dyslipidemia with and without hyper-low-density lipoprotein-cholesterolemia was 9.7%, 41.0%, 63.8%, and 19.5%, respectively, and the prevalence of these conditions increased with increasing obesity. The risk for diabetes and hypertension was increased markedly (odds ratios of 12.95 and 19.44, respectively), and that for dyslipidemia with and without hyper-low-density lipoprotein-cholesterolemia was modestly increased (odds ratios of 2.59 and 3.65, respectively) at a BMI of ≥ 35 kg/m^2^ compared with normal weight. Analysis by gender revealed that the obesity-related risk for dyslipidemia with hyper-low-density lipoprotein-cholesterolemia was small compared with other comorbidities in women, while the risk for all comorbidities elevated similarly in men. Our results suggest the importance of public health intervention for obesity to suppress its comorbidities, especially diabetes and hypertension, at this age.

## Introduction

Despite its established health risk and economic burden, the prevalence of obesity is increasing globally^1^. In 2014, ~ 266 million men and ~ 375 million women were obese worldwide^2^. In Japan, ~ 30% of men and ~ 20% of women are currently classified as obese, defined as a body mass index (BMI) of ≥ 25 kg/m^2,3^.

Obesity is associated with numerous comorbidities—including hypertension, type 2 diabetes mellitus, dyslipidemia, hyperuricemia, nonalcoholic steatohepatitis, obstructive sleep apnea, osteoarthritis, and reproductive problems^4^—among which hypertension, diabetes, and dyslipidemia are the most common obesity-related conditions and are closely associated with the development of cardiovascular disease and consequent increased mortality^5^. It is therefore important to clarify the impact of obesity on these three conditions for overall health care and with regard to its implications for the treatment of obesity.

Skeletal muscle mass declines at an increasing rate after the age of 65 years^6^, leading to sarcopenia and its associated increased risk of all-cause mortality^7^. Not only obesity but also the condition of underweight is therefore a concern for individuals aged 65 years and older, indicating the importance of body weight management for this age group. We have now investigated the prevalence of the three key obesity-related diseases—diabetes, hypertension, and dyslipidemia—and the risk for their development according to weight status in a population of 65-year-old individuals undergoing health checkups in Kobe, Japan.

## Materials and methods

### Study population

This cross-sectional study included 10,852 participants who were 65-year-old members of National Health Insurance, one of the public medical insurance systems in Japan. They underwent Specific Health Checkups (assessments for the prevention of lifestyle-related diseases) implemented by Kobe City between April 2017 and March 2021. In these checkups, physicians interviewed the participants and conducted physical examinations.

### Data collection

Height, weight, waist circumference, and blood pressure were measured for all participants. Blood was also collected for analysis of lipid metabolism (serum concentrations of triglyceride [TG], high-density lipoprotein-cholesterol [HDL-C], and low-density lipoprotein-cholesterol [LDL-C]), glucose metabolism (fasting plasma glucose concentration [FPG] and glycated hemoglobin [HbA_1c_] level), liver function (serum levels of aspartate aminotransferase [AST], alanine aminotransferase [ALT], and γ-glutamyl transpeptidase [γ-GTP]), and renal function (serum creatinine concentration and estimated glomerular filtration rate [eGFR]). Medications, smoking status, and physical activity were also investigated with a questionnaire at the checkups. With regard to physical activity, exercise habit was determined on the basis either of engagement in sweat-inducing exercise for > 30 min on at least 2 days a week over 1 year or of taking a walk or undertaking equivalent physical activity for > 1 h each day. These questions are also included in a standardized questionnaire for Specific Health Checkups developed by the Japanese Ministry of Health, Labor, and Welfare for the assessment of physical activity.

### Definitions of obesity, diabetes mellitus, hypertension, dyslipidemia, and metabolic syndrome

Underweight, normal weight, and obesity were respectively defined according to the diagnostic criteria of the Japan Society for the Study of Obesity^8^ as: BMI < 18.5 kg/m^2^, 18.5 kg/m^2^ ≤ BMI < 25 kg/m^2^, and BMI ≥ 25 kg/m^2^. In addition, obesity was classified into three groups: class I (25 kg/m^2^ ≤ BMI < 30 kg/m^2^), class II (30 kg/m^2^ ≤ BMI < 35 kg/m^2^), and class III (BMI ≥ 35 kg/m^2^). Diabetes mellitus was diagnosed according to the diagnostic criteria of the Japan Diabetes Association^9^ as an FPG of ≥ 126 mg/dL and HbA_1c_ level of ≥ 6.5%. Hypertension was defined according to the guidelines of the Japanese Society of Hypertension 2019^10^ as a systolic blood pressure (SBP) of ≥ 140 mmHg or diastolic blood pressure (DBP) of ≥ 90 mmHg (or both). Concerning dyslipidemia, two definitions were adopted. One was defined according to the diagnostic criteria of the Japan Atherosclerosis Society^11^ as a TG concentration of ≥ 150 mg/dL, HDL-C concentration of < 40 mg/dL, or LDL-C concentration of ≥ 140 mg/dL (dyslipidemia (TG/HDL/LDL)). Dyslipidemia characterized by hypertriglyceridemia and hypo-HDL-cholesterolemia is known to be closely associated with metabolic syndrome and atherosclerosis^12^. The other was therefore defined as a TG concentration of ≥ 150 mg/dL or HDL-C concentration of < 40 mg/dL (dyslipidemia (TG/HDL)). Participants undergoing treatment with antihypertensive, lipid-lowering, or hypoglycemic agents as revealed from the questionnaire were also defined as having hypertension, dyslipidemia, or diabetes, respectively. Dyslipidemia (TG/HDL) was however determined only by TG and HDL-C concentrations regardless of the presence or absence of medication because the details of lipid-lowering drugs were unknown in this study. Metabolic syndrome was basically diagnosed by the Japanese definition published by the Committee to Evaluate Diagnostic Standards for Metabolic Syndrome in 2005^13^. In particular, abdominal adiposity (waist circumference of ≥ 85 cm in men and of ≥ 90 cm in women) was a required condition. Moreover, the presence of two or more of the following three abnormalities was necessary: hypertension (blood pressure of ≥ 130/85 mmHg or participants with antihypertensive agents), glucose intolerance (fasting glucose levels of ≥ 110 mg/dL or participants with hypoglycemic agents), and dyslipidemia (TG levels of ≥ 150 mg/dL, HDL-C levels of < 40 mg/dL, or participants treated with lipid-lowering agents).

### Statistical analysis

Data are presented as the mean ± SD for parameters with a normal distribution. Differences in continuous or noncontinuous variables between sexes were evaluated with Student’s *t* test and the Mann–Whitney U test, respectively. The relation between obesity grade and either continuous variables or noncontinuous variables and disease prevalence was examined with the Jonckheere–Terpstra trend test and the Armitage trend test, respectively. Binomial logistic regression analysis was adopted for calculation of the odds ratio (OR) and its 95% confidence interval (CI) with regard to disease prevalence according to obesity grade; the results were adjusted for sex, smoking status, and exercise habit. Intergroup comparisons of the OR for each disease were adjusted for multiplicity by Bonferroni’s correction. In calculating OR in the classification of BMI by gender, all participants in the class III obesity group in men had hypertension, therefore, they were eliminated from the analysis target. A *P* value of < 0.05 was considered statistically significant. All statistical analysis was performed with JMP software version 16.0.

### End points

The primary end point of the study was obesity-related risk for the presence of hypertension, dyslipidemia, and diabetes in individuals aged 65 years. The secondary end point was the prevalence of the three conditions according to obesity grade in the same population.

### Statement of ethics

The study conformed to the provisions of the Declaration of Helsinki (revised in 2013) and was approved by the ethics committees of Kobe University Graduate School of Medicine on March 18, 2022 (Approval No. B210250) and of Kobe City on April 7, 2022 (Approval No. 3–10). All participants who underwent Specific Health Checkups provided written informed consent for the use of their data in the checkups for analysis, and their anonymity was preserved. Potential participants were given the opportunity to decline participation or opt out from the study.

## Results

### Characteristics of the study participants

The characteristics of the study participants are shown in Table 1. A total of 10,852 individuals (3576 men and 7276 women) aged 65 years was enrolled in the study. The mean BMI for all participants was 22.4 ± 3.4 kg/m^2^. All parameters differed significantly between men and women. The prevalence of obesity and metabolic syndrome was 20.6% (2233/10,852) and 14.9% (1615/10,852) among all participants, 31.4% (1124/3576) and 30.6% (1096/3576) among men, and 15.2% (1109/7276) and 7.1% (519/7276) among women, respectively.

**Table 1:** Characteristics of the study participants.

Characteristic	Total (*n* = 10,852)	Male (*n* = 3576)	Female (*n* = 7276)	*P*
Body weight (kg)	57.3 ± 11.3	67.1 ± 10.1	52.5 ± 8.3	< 0.001
BMI (kg/m^2^)	22.4 ± 3.4	23.7 ± 3.2	21.8 ± 3.4	< 0.001
Waist circumference (cm)	81.9 ± 9.7	85.8 ± 8.9	79.9 ± 9.5	< 0.001
SBP (mmHg)	127.0 ± 17.7	130.3 ± 17.4	125.4 ± 17.6	< 0.001
DBP (mmHg)	76.2 ± 11.2	79.9 ± 11	74.4 ± 10.8	< 0.001
Creatinine (mg/dL)	0.72 ± 0.24	0.87 ± 0.34	0.64 ± 0.11	< 0.001
eGFR (mL min^−1^ 1.73 m^–2^)	72.5 ± 13.5	71.4 ± 14.0	73.0 ± 13.3	0.014
AST (IU/L)	23.3 ± 9.1	24.7 ± 11.7	22.6 ± 7.4	< 0.001
ALT (IU/L)	20.3 ± 12.6	23.7 ± 14.7	18.7 ± 11.1	< 0.001
γ-GTP (IU/L)	33.9 ± 41.3	48.7 ± 61.0	26.6 ± 26.6	< 0.001
LDL-C (mmol/L)	3.37 ± 0.83	3.15 ± 0.82	3.48 ± 0.81	< 0.001
HDL-C (mmol/L)	1.78 ± 0.47	1.55 ± 0.43	1.89 ± 0.45	< 0.001
TG (mmol/L)	1.26 ± 0.97	1.49 ± 1.41	1.14 ± 0.63	< 0.001
FPG (mmol/L)	5.46 ± 1.08	5.78 ± 1.27	5.29 ± 0.93	< 0.001
HbA_1c_ (mmol/mol)	39.2 ± 6.5	40.1 ± 8.0	38.8 ± 5.5	< 0.001
Number of comorbidities	1.15 ± 0.81	1.31 ± 0.86	1.06 ± 0.77	< 0.001
Smoking rate, *n*, (%)	1114 (10.3)	784 (21.9)	330 (4.5)	< 0.001
Presence of exercise habit, *n*, (%)	4885 (45.0)	1656 (46.3)	3229 (44.4)	< 0.001
Medications				
Hypoglycemic drugs, *n*, (%)	568 (5.2)	315 (8.8)	253 (3.5)	< 0.001
Antihypertensive drugs, *n*, (%)	2829 (26.1)	1301 (36.4)	1528 (21.0)	< 0.001
Lipid-lowering drugs, *n*, (%)	2611 (24.1)	777 (21.7)	1834 (25.2)	< 0.001
Metabolic syndrome, *n*, (%)	1615 (14.9)	1096 (30.6)	519 (7.1)	< 0.001

Data are means ± SD or *n* (%). All abbreviations are defined in the text. *P* values for comparisons between males and females were calculated with Student’s *t* test or the Mann–Whitney U test as appropriate.

### Clinical variables according to BMI category

The prevalence of underweight, normal weight, and obesity (classes I–III) among all participants was 10.2%, 69.2%, and 20.6%, respectively (Table 2). Severe obesity with a BMI of ≥ 35 kg/m^2^ (obesity class III) was present in only 0.37% of all participants. All clinical parameters with the exception of serum LDL-C level differed significantly across BMI categories. Waist circumference, blood pressure, serum creatinine concentration, serum AST, ALT, and γ-GTP levels, serum TG concentration, FPG, and HbA_1c_ level increased with obesity, whereas serum HDL-C concentration decreased with it (Table 2). With regard to medications, prescriptions for diabetes, hypertension, and dyslipidemia increased with increasing obesity. The prevalence of metabolic syndrome increased with obesity from 0% (0/1,105) at underweight group to 85% (34/40) at class III obesity group. The number of comorbidities (diabetes, hypertension, and dyslipidemia (TG/HDL/LDL)) per individual also increased with obesity. In the analysis by gender, serum creatinine concentration, eGFR, serum AST level, serum LDL-C level, and smoking status did not differ across BMI categories in women, whereas all clinical parameters differed significantly in men (Table 3).

**Table 2:** Characteristics of the study participants according to BMI category.

Characteristic	BMI category					
	Under-weight (*n* = 1105, 10.2%)	Normal-weight (*n* = 7514, 69.2%)	Obesity (*n* = 2233, 20.6%)			*P*
			Class I (*n* = 1932, 17.8%)	Class II (*n* = 261, 2.4%)	Class III (*n* = 40, 0.37%)	
Male, *n,* (%)	127 (11.5)	2325 (30.9)	1004 (52.0)	100 (38.3)	20 (50.0)	< 0.001
Body weight (kg)	43.1 ± 4.3	55.2 ± 7.7	69.7 ± 8.4	80.2 ± 9.2	97.2 ± 12.7	< 0.001
BMI (kg/m^2^)	17.3 ± 1.0	21.7 ± 1.8	26.7 ± 1.3	31.7 ± 1.3	37.3 ± 2.2	< 0.001
Waist circumference (cm)	68.7 ± 5.1	80.2 ± 6.5	92.4 ± 5.8	103.5 ± 6.0	116.3 ± 9.9	< 0.001
SBP (mmHg)	119.4 ± 18.1	126.0 ± 17.3	133.6 ± 16.6	136.2 ± 15.0	140.9 ± 17.3	< 0.001
DBP (mmHg)	71.6 ± 11.5	75.6 ± 10.8	80.3 ± 10.8	81.4 ± 9.6	81.5 ± 14.4	< 0.001
Creatinine (mg/dL)	0.66 ± 0.13	0.71 ± 0.25	0.77 ± 0.22	0.74 ± 0.29	0.77 ± 0.21	< 0.001
eGFR (mL min^−1^ 1.73 m^–2^)	73.5 ± 12.8	72.6 ± 13	71.4 ± 15.3	72.7 ± 15.6	71.3 ± 15.3	< 0.001
AST (IU/L)	23.1 ± 6.6	22.6 ± 8.5	24.9 ± 10.8	29.8 ± 15.5	28.4 ± 13.9	< 0.001
ALT (IU/L)	16.5 ± 6.6	18.9 ± 10.7	26.1 ± 15.9	35.0 ± 25.0	31.5 ± 18.1	< 0.001
γ-GTP (IU/L)	26.4 ± 29.4	32.3 ± 41.5	42.1 ± 44.7	46.4 ± 39.4	48.2 ± 33.3	< 0.001
LDL-C (mmol/L)	3.29 ± 0.79	3.40 ± 0.82	3.33 ± 0.87	3.20 ± 0.88	3.11 ± 0.78	0.082
HDL-C (mmol/L)	2.13 ± 0.46	1.81 ± 0.46	1.51 ± 0.37	1.49 ± 0.34	1.26 ± 0.26	< 0.001
TG (mmol/L)	0.91 ± 0.52	1.20 ± 0.74	1.62 ± 1.61	1.68 ± 1.11	1.89 ± 0.84	< 0.001
FPG (mmol/L)	5.13 ± 0.80	5.37 ± 0.97	5.88 ± 1.36	6.16 ± 1.39	7.12 ± 2.21	< 0.001
HbA_1c_ (mmol/mol)	37.6 ± 4.52	38.7 ± 5.95	41.3 ± 7.85	43.7 ± 9.04	47.7 ± 11.6	< 0.001
Number of comorbidities	0.70 ± 0.68	1.06 ± 0.76	1.59 ± 0.80	1.93 ± 0.74	2.25 ± 0.74	< 0.001
Smoking rate, *n*, (%)	84 (7.6)	754 (10.0)	248 (12.8)	25 (9.6)	3 (7.5)	< 0.001
Presence of exercise habit, *n*, (%)	493 (44.7)	3507 (46.7)	779 (40.3)	100 (38.3)	6 (15.0)	< 0.001
Medications						
Hypoglycemic drugs, *n*, (%)	13 (1.2)	282 (3.8)	207 (10.7)	52 (19.9)	14 (35.0)	< 0.001
Antihypertensive drugs, *n*, (%)	117 (10.6)	1615 (21.5)	891 (46.1)	174 (66.7)	32 (80.0)	< 0.001
Lipid-lowering drugs, *n*, (%)	154 (13.9)	1666 (22.2)	660 (34.2)	113 (43.3)	18 (45.0)	< 0.001
Metabolic syndrome, *n*, (%)	0 (0)	492 (6.6)	901 (46.6)	188 (72.0)	34 (85.0)	< 0.001

Data are means ± SD or *n* (%). All abbreviations are defined in the text. *P* values were calculated with the Jonckheere–Terpstra trend test or the Armitage trend test as appropriate.

**Table 3:** Characteristics of study participants by BMI category.

(a) Male						
Variables	BMI category					
	Under-weight (*n* = 127, 3.6%)	Normal-weight (*n* = 2325, 65.0%)	Obesity (*n* = 1124, 31.4%)			*P*
			Class I (*n* = 1004, 28.1%)	Class II (*n* = 100, 2.8%)	Class III (*n* = 20, 0.6%)	
Body weight (kg)	49.0 ± 4.2	63.2 ± 6.3	75.4 ± 6.3	88.8 ± 6.5	106.5 ± 8.0	< 0.001
BMI (kg/m^2^)	17.3 ± 1.1	22.3 ± 1.6	26.8 ± 1.3	31.7 ± 1.3	37.1 ± 1.8	< 0.001
Waist circumference (cm)	69.4 ± 4.4	82.5 ± 5.8	93.2 ± 5.3	104.8 ± 5.9	118.3 ± 7.8	< 0.001
SBP (mmHg)	122.8 ± 16.8	128.4 ± 17.3	134.9 ± 16.8	135.6 ± 15.5	142.5 ± 15.1	< 0.001
DBP (mmHg)	75.1 ± 11.7	78.8 ± 10.7	82.7 ± 10.9	83.1 ± 10.6	83.5 ± 15.6	< 0.001
Creatinine (mg/dL)	0.81 ± 0.18	0.87 ± 0.38	0.89 ± 0.22	0.91 ± 0.37	0.89 ± 0.19	< 0.001
eGFR (mL min^–1^ 1.73 m^–2^)	77.7 ± 16.8	72.0 ± 13.6	69.3 ± 13.6	70.6 ± 17.5	70.1 ± 14.8	< 0.001
AST (IU/L)	24.5 ± 11.0	23.8 ± 11.7	26.1 ± 11.2	30.6 ± 16.2	29.1 ± 10.9	< 0.001
ALT (IU/L)	17.8 ± 9.0	21.3 ± 12.8	28.6 ± 16.2	36.8 ± 22.8	32.8 ± 17.9	< 0.001
γGTP (IU/L)	40.2 ± 51.8	46.5 ± 64.2	53.8 ± 55.3	58.6 ± 48.9	59.9 ± 39.3	< 0.001
LDL-C (mmol/L)	2.77 ± 0.78	3.16 ± 0.81	3.18 ± 0.83	3.16 ± 0.83	2.93 ± 0.85	0.019
HDL-C (mmol/L)	1.92 ± 0.49	1.62 ± 0.44	1.39 ± 0.33	1.29 ± 0.28	1.20 ± 0.22	< 0.001
TG (mmol/L)	1.07 ± 0.99	1.36 ± 0.96	1.80 ± 2.10	2.03 ± 1.45	2.10 ± 0.97	< 0.001
Fasting plasma glucose (mmol/L)	5.62 ± 1.43	5.65 ± 1.17	6.04 ± 1.36	6.37 ± 1.24	7.22 ± 2.40	< 0.001
HbA_1c_ (mmol/mol)	38.5 ± 7.2	39.1 ± 7.6	41.7 ± 8.1	44.8 ± 9.9	49.3 ± 14.2	< 0.001
Number of comorbidities	0.67 ± 0.74	1.15 ± 0.82	1.68 ± 0.79	2.10 ± 0.66	2.40 ± 0.68	< 0.001
Smoking rate, *n*, (%)	32 (25.2)	535 (23.0)	200 (19.9)	15 (15.0)	2 (10.0)	0.001
Physical activity, *n*, (%)	51 (40.2)	1164 (50.1)	406 (40.4)	32 (32.0)	3 (15.0)	< 0.001
Medication						
Hypoglycemic drugs, *n*, (%)	7 (5.5)	154 (6.6)	124 (12.4)	25 (25.0)	5 (25.0)	< 0.001
Antihypertensive drugs, *n*, (%)	19 (15.0)	665 (28.6)	530 (52.8)	71 (71.0)	16 (80.0)	< 0.001
Lipid-lowering drugs, *n*, (%)	8 (6.3)	421 (18.1)	300 (29.9)	37 (37.0)	11 (55.0)	< 0.001
Metabolic syndrome, *n*, (%)	0 (0.0)	385 (16.6)	612 (61.0)	81 (81.0)	18 (90.0)	< 0.001

(b) Female						
Variables	BMI category					
	Under-weight (*n* = 978, 13.4%)	Normal-weight (*n* = 5189, 71.3%)	Obesity (*n* = 1109, 15.2%)			
			Class I (*n* = 928, 12.8%)	Class II (*n* = 161, 2.2%)	Class III (*n* = 20, 0.3%)	*P*
Body weight (kg)	42.3 ± 3.6	51.7 ± 5.1	63.4 ± 5.3	74.9 ± 6.1	87.9 ± 9.2	< 0.001
BMI (kg/m^2^)	17.3 ± 1.0	21.4 ± 1.7	26.7 ± 1.3	31.7 ± 1.4	37.6 ± 2.5	< 0.001
Waist circumference (cm)	68.6 ± 5.1	79.1 ± 6.5	91.6 ± 6.1	102.8 ± 5.9	114.3 ± 11.5	< 0.001
SBP (mmHg)	118.9 ± 18.3	125.0 ± 17.1	132.3 ± 16.2	136.6 ± 14.7	139.2 ± 19.5	< 0.001
DBP (mmHg)	71.1 ± 11.4	74.2 ± 10.5	77.8 ± 10.1	80.4 ± 8.8	79.5 ± 13.2	< 0.001
Creatinine (mg/dL)	0.64 ± 0.10	0.64 ± 0.11	0.64 ± 0.11	0.64 ± 0.14	0.66 ± 0.16	0.735
eGFR (mL min^−1^ 1.73 m^−2^)	73.0 ± 12.2	72.8 ± 12.7	73.5 ± 16.7	74.1 ± 14.3	72.4 ± 16.0	0.722
AST (IU/L)	22.9 ± 5.8	22.1 ± 6.6	23.7 ± 10.2	29.3 ± 15.0	27.7 ± 16.7	0.636
ALT (IU/L)	16.3 ± 6.3	17.7 ± 9.5	23.4 ± 15.0	34.0 ± 26.3	30.2 ± 18.7	< 0.001
γGTP (IU/L)	24.7 ± 24.5	25.7 ± 22.7	31.8 ± 24.4	38.8 ± 29.8	36.5 ± 21.2	< 0.001
LDL-C (mmol/L)	3.35 ± 0.77	3.50 ± 0.80	3.50 ± 0.87	3.22 ± 0.91	3.29 ± 0.68	0.123
HDL-C (mmol/L)	2.15 ± 0.45	1.89 ± 0.44	1.65 ± 0.36	1.62 ± 0.32	1.33 ± 0.28	< 0.001
TG (mmol/L)	0.88 ± 0.41	1.13 ± 0.61	1.42 ± 0.76	1.47 ± 0.75	1.68 ± 0.64	< 0.001
Fasting plasma glucose (mmol/L)	5.06 ± 0.65	5.24 ± 0.82	5.69 ± 1.34	6.04 ± 1.47	7.00 ± 2.05	< 0.001
HbA_1c_ (mmol/mol)	37.5 ± 4.0	38.4 ± 5.0	40.9 ± 7.6	43.0 ± 8.4	46.1 ± 8.2	< 0.001
Number of comorbidities	0.71 ± 0.67	1.03 ± 0.73	1.49 ± 0.78	1.83 ± 0.77	2.10 ± 0.79	< 0.001
Smoking rate, *n*, (%)	52 (5.3)	219 (4.2)	48 (5.2)	10 (6.2)	1 (5.0)	0.358
Physical activity, *n*, (%)	442 (45.2)	2343 (45.2)	373 (40.2)	68 (42.2)	3 (15.0)	0.003
Medication						
Hypoglycemic drugs, *n*, (%)	6 (0.6)	128 (2.5)	83 (8.9)	27 (16.8)	9 (45.0)	< 0.001
Antihypertensive drugs, *n*, (%)	98 (10.0)	950 (18.3)	361 (38.9)	103 (64.0)	16 (80.0)	< 0.001
Lipid-lowering drugs, *n*, (%)	146 (14.9)	1245 (24.0)	360 (38.8)	76 (47.2)	7 (35.0)	< 0.001
Metabolic syndrome, *n*, (%)	0 (0.0)	107 (2.1)	289 (31.1)	107 (66.5)	16 (80.6)	< 0.001

Data are mean ± SD or *n* (%). All abbreviations are defined in the text. *P* values were calculated with the Jonckheere–Terpstra trend test or the Armitage trend test as appropriate.

### Prevalence of diabetes, hypertension, and dyslipidemia according to BMI category

**Figure 1:**
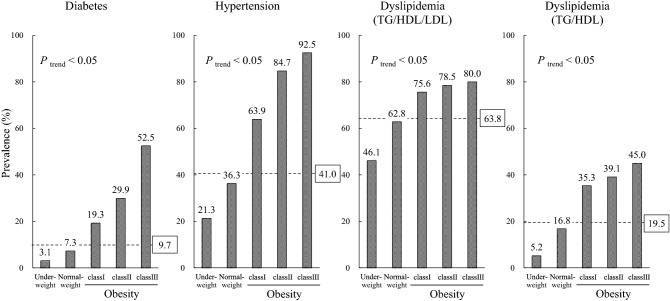
Prevalence of diabetes, hypertension, and dyslipidemia according to BMI classification. Numbers above bars represent prevalence (%). *P*_trend_ < 0.05 indicates a significant difference in disease prevalence among BMI categories as assessed by the Armitage trend test. The numbers in boxes indicate the prevalence of each disease among all participants. Dyslipidemia (TG/HDL/LDL) and dyslipidemia (TG/HDL) were defined in the text.

**Figure 2:**
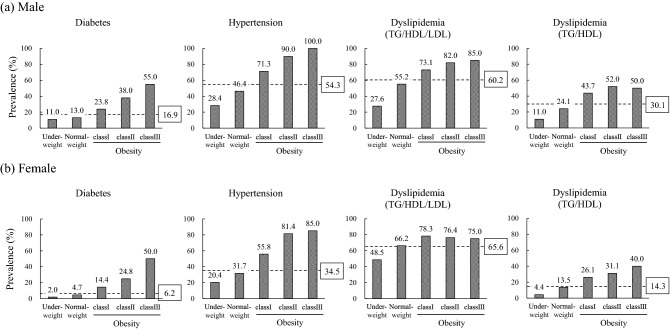
Prevalence of diabetes, hypertension, and dyslipidemia according to BMI classification by gender. Numbers above bars represent prevalence (%). All analyses showed a significant difference in disease prevalence among BMI categories as assessed by the Armitage trend test. The numbers in boxes indicate the prevalence of each disease among each sex of participants. Dyslipidemia (TG/HDL/LDL) and dyslipidemia (TG/HDL) were defined in the text.

The prevalence of diabetes, hypertension, dyslipidemia (TG/HDL/LDL), and dyslipidemia (TG/HDL) among all participants was 9.7%, 41.0%, 63.8%, and 19.5%, respectively (Fig. 1). Diabetes had the lowest prevalence of the three diseases in each BMI category excluding class III obesity group in dyslipidemia (TG/HDL). Dyslipidemia (TG/HDL/LDL) showed the highest prevalence in underweight, normal weight, and class I obesity groups, whereas hypertension showed the highest prevalence in class II and class III obesity groups. The prevalence of diabetes, hypertension, and dyslipidemia (TG/HDL/LDL) increased with increasing BMI, with that of diabetes and hypertension increasing markedly and that of dyslipidemia to a lesser extent. The prevalence of dyslipidemia (TG/HDL) also increased with obesity and more evidently than dyslipidemia (TG/HDL/LDL). In the analysis by gender, the prevalence of diabetes, hypertension, and dyslipidemia (TG/HDL) was substantially higher in men than in women, whereas that of dyslipidemia (TG/HDL/LDL) was slightly but significantly higher in women (Fig. 2). The prevalence of diabetes, hypertension, and dyslipidemia (TG/HDL/LDL) was increased with increasing obesity in men. In women, however, the prevalence of dyslipidemia (TG/HDL/LDL) peaked in class I obesity group, whereas that of dyslipidemia (TG/HDL) was increased with obesity.

### Risk for diabetes, hypertension, and dyslipidemia conferred by obesity

**Figure 3:**
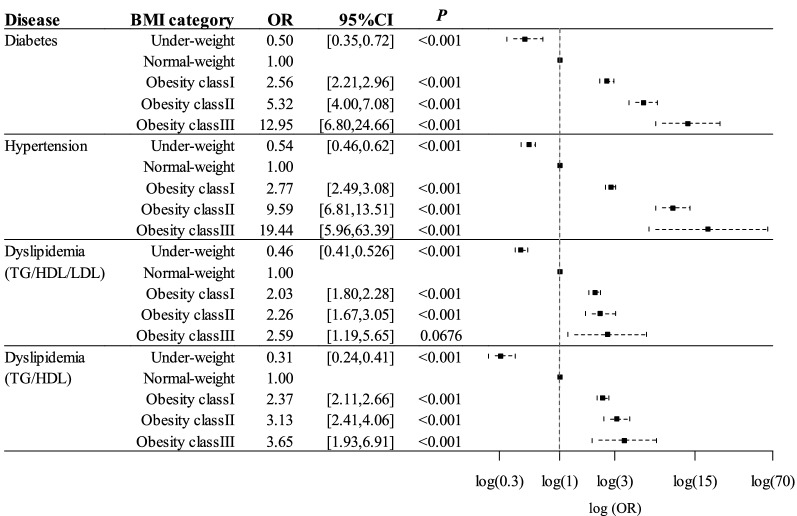
Adjusted OR and its 95% CI for diabetes, hypertension, and dyslipidemia in participants of each BMI category with normal weight as the reference. Each OR was adjusted for sex, smoking status, and exercise habit. The horizontal axis represents the log-transformed adjusted OR. *P*-values were calculated in the comparison of the OR for each disease between normal-weight group (reference) and other groups by adjusting for multiplicity by Bonferroni’s correction. Dyslipidemia (TG/HDL/LDL) and dyslipidemia (TG/HDL) were defined in the text.

**Figure 4:**
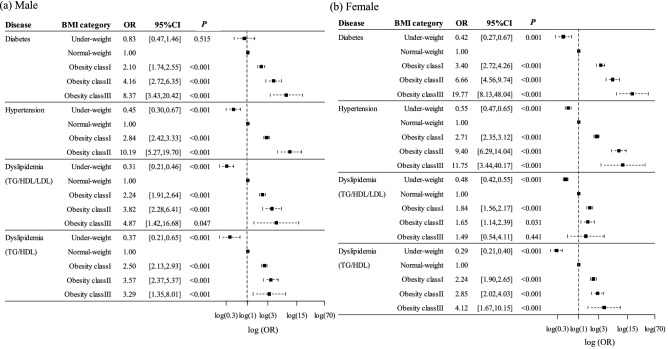
Adjusted OR and its 95% CI for diabetes, hypertension, and dyslipidemia in participants of each BMI category by gender with normal weight as the reference. Each OR was adjusted for smoking status and exercise habit. The horizontal axis represents the log-transformed adjusted OR. *P*-values were calculated in the comparison of the OR for each disease between normal-weight group (reference) and other groups by adjusting for multiplicity by Bonferroni’s correction. Dyslipidemia (TG/HDL/LDL) and dyslipidemia (TG/HDL) were defined in the text.

The increased prevalence for each condition associated with obesity was calculated by binomial logistic regression analysis after adjustment for sex, smoking status, and physical activity (Fig. 3). The OR for diabetes in underweight and obesity class I, II, and III groups was 0.50, 2.56, 5.32, and 12.95, respectively, with normal weight as the reference, and it increased significantly with increasing BMI category. The OR for hypertension in the same categories was 0.54, 2.77, 9.59, and 19.44, respectively, and it also increased significantly with increasing BMI. The corresponding OR values for dyslipidemia (TG/HDL/LDL) were 0.46, 2.03, 2.26, and 2.59. The increase in the OR for this type of dyslipidemia with BMI was thus less pronounced than was that for diabetes or hypertension, and there was no intercategory difference in the OR for dyslipidemia between normal weight and class III obesity groups. The corresponding OR values were 0.31, 2.37, 3.13, and 3.65, which increased with increasing BMI categories in dyslipidemia (TG/HDL). In the analysis by gender, the OR for diabetes and hypertension increased markedly and that for dyslipidemia (TG/HDL) increased moderately with obesity in both men and women (Fig. 4). Concerning dyslipidemia (TG/HDL/LDL), the OR for it increased moderately in men (OR of 4.87), while it increased to a lesser extent and peaked in the class I obesity group in women (OR of 1.84).

### Analysis of the prevalence and risk when obesity is classified by waist circumference

Abdominal fat is closely associated with insulin resistance-related comorbidities^14^. We therefore assessed the relation between obesity and comorbidities by classifying the participants with a quintile of waist circumference which well reflects abdominal fat mass^15^. Only serum LDL-C level remained unchanged across waist circumference categories among all clinical parameters (Supplementary Table S1 online). The prevalence of each condition increased with increasing waist circumference with the largest waist circumference category being the highest (Supplementary Fig. S1 online). The ORs comparing the highest to the lowest waist circumference categories for diabetes, hypertension, dyslipidemia (TG/HDL/LDL), and dyslipidemia (TG/HDL) were 7.11, 5.05, 3.82, and 7.81, respectively (Supplementary Fig. S2 online). In the analysis by gender, the characteristics of participants are shown in Supplementary Table S2 online. The prevalence of each condition increased with increasing waist circumference in both genders (Supplementary Fig. S3 online). The OR for all comorbidities increased similarly across waist circumference with the largest waist circumference category being the highest in both genders (Supplementary Fig. S4 online).

## Discussion

We have here found that the prevalence of diabetes, hypertension, and dyslipidemia increased significantly with increasing BMI category. A higher than normal BMI was an independent risk factor for the three diseases even after adjustment for sex, smoking status, and physical activity, with the risk conferred by obesity being substantially higher for diabetes and hypertension than for dyslipidemia. Such a tendency of the risk due to obesity was more prominent in women compared with men. As far as we are aware, our study is the first to examine the prevalence of the three major obesity-related comorbidities by BMI category as well as the risk for the presence of each of these conditions due to obesity by gender in Japanese individuals of this age.

A distinctive finding of the present study is that obesity was associated with a high risk for the presence of diabetes and hypertension. In general, the obesity-related risk for diabetes and hypertension has been found to be higher than that for dyslipidemia in economically wealthy countries^16–19^. A study of Japanese individuals aged 18–85 years thus also found that the obesity-related risk for diabetes and hypertension was much higher than that for dyslipidemia, although the ages of the participants differed between this previous study and our study^16^. It may therefore be a characteristic of Japanese that the development of diabetes and hypertension is closely associated with increasing obesity. On the other hand, a study of Kenyan adults showed that obesity was associated with hypertension and dyslipidemia but not with diabetes^20^. This difference may reflect the fact that most individuals with diabetes are younger and leaner in sub-Saharan Africa compared with those in high-income countries^21^.

Japanese individuals have a decreased capacity for insulin secretion even during periods of normal glucose tolerance compared with white individuals^22^. In addition, Japanese have more abdominal visceral fat relative to subcutaneous fat than do whites^23^. Visceral fat is closely associated with insulin resistance, which contributes to the development of diabetes and hypertension^24–26^. These various observations may explain why the development of diabetes and hypertension increases markedly with increasing obesity in Japanese.

We found that the increase in the prevalence of dyslipidemia (TG/HDL/LDL) according to BMI category was less pronounced compared with that for diabetes or hypertension. In addition, the OR for dyslipidemia (TG/HDL/LDL) did not show large differences among obesity classes I to III. Previous studies have also shown that the prevalence of dyslipidemia peaks in association with moderate obesity rather than with severe obesity^12–14,27^. These findings thus show that dyslipidemia is the least affected by obesity of the three major comorbidities. Dyslipidemia induced by obesity is generally characterized by a high serum concentration of TG, a decreased HDL-C concentration, and an optimal or slightly increased level of LDL-C^28^. In the present study, there was no significant difference in serum LDL-C levels across BMI categories in total subjects and in women, suggesting that BMI did not affect serum LDL-cholesterol concentration. This may have limited the effect of obesity on the risk for dyslipidemia (TG/HDL/LDL) especially in women, given that dyslipidemia (TG/HDL/LDL) is defined by hypertriglyceridemia, hypo-HDL-cholesterolemia, or hyper-LDL-cholesterolemia. In fact, the risk of obesity for dyslipidemia (TG/HDL) increased with obesity more prominently than dyslipidemia (TG/HDL/LDL) in women (Fig. 4). Previous studies have not obtained consistent results concerning the relation between weight loss and improvement in LDL-C levels^29–31^.

In the present study, underweight participants showed a lower risk for diabetes, hypertension, and dyslipidemia compared with those of normal weight. On the other hand, a low BMI is a risk factor for sarcopenia, which is associated with frailty in 65-year-old Japanese^32^, and the prevalence of this condition was previously found to be ~ 20% in underweight individuals with a BMI of < 17.0 kg/m^2,32^. Sarcopenia is also associated with an increased risk of all-cause mortality^7^. All-cause mortality is minimal for individuals with a BMI of 20.0–25.0 kg/m^2^ irrespective of ethnicity^33^. These observations suggest that being underweight should not be recommended for elderly individuals even if it is associated with a lower risk for diabetes, hypertension, and dyslipidemia. Individualized and well-balanced health guidance is thus necessary for 65-year-olds in order to prevent both obesity-related diseases and sarcopenia.

The main limitation of our study is that all participants were 65 years of age. However, this can also be viewed as an advantage in that the effects of obesity on the presence of comorbidities can be studied specifically at this age. Secondly, we couldn’t select the participants with hyper-LDL-cholesterolemia only, given that we did not know whether lipid-lowering drugs were prescribed for hyper-LDL-cholesterolemia or hypertriglyceridemia. Another limitation is that there were few participants with severe obesity, which resulted in large 95% CIs of the ORs for class III obesity in the classification by BMI. Our study was also performed with residents of only one large city in Japan, with the consequence that it remains to be established whether our findings apply to individuals in other regions of Japan or other countries. Finally, the cross-sectional design of the study makes it difficult to establish the causal relation between obesity and the three examined diseases.

## Conclusion

In conclusion, the prevalence of diabetes, hypertension, and dyslipidemia increased significantly with increasing BMI in 65-year-old Japanese individuals, and a higher BMI was found to be an independent risk factor for these diseases, with the effects on diabetes and hypertension being larger than that on dyslipidemia.

## Supplementary Information

Supplementary Information.

## Data Availability

The data that support the findings of this study are not publicly available because of provisions of the Kobe City government. Further enquiries can be directed to the corresponding author.

## Abbreviations

BMI: Body mass index
TG: Triglyceride
HDL-C: High-density lipoprotein–cholesterol
LDL-C: Low-density lipoprotein–cholesterol
FPG: Fasting plasma glucose concentration
HbA1c: Glycated hemoglobin
AST: Aspartate aminotransferase
ALT: Alanine aminotransferase
γ-GTP: γ-Glutamyl transpeptidase
eGFR: Estimated glomerular filtration rate
SBP: Systolic blood pressure
DBP: Diastolic blood pressure
OR: Odds ratio
CI: Confidence interval
