# Supplementary material for: Obesity and risk for its comorbidities diabetes, hypertension, and dyslipidemia in Japanese individuals aged 65 years
Source: Sci Rep. 2023 Feb 9;13:2346. doi: 10.1038/s41598-023-29276-7 (PMC9911391; doi:10.1038/s41598-023-29276-7)
Supplement: Supplementary file 1 — Supplementary Information. [file 41598_2023_29276_MOESM1_ESM.pdf]

Supplementary Information

Obesity and Risk for Its Comorbidities Diabetes, Hypertension, and Dyslipidemia in Japanese Individuals Aged 65 Years

Tomoko Yamada, Maki Kimura-Koyanagi, Kazuhiko Sakaguchi, Wataru Ogawa, *Yoshikazu Tamori.

* Corresponding author: Yoshikazu Tamori

Division of Creative Health Promotion, Department of Social/Community Medicine and Health Science, Kobe University Graduate School of Medicine, 7-5-1 Kusunoki-cho, Chuo-ku, Kobe 650-0017, Japan.

Tel.: +81-78-382-5861. Fax: +81-78-382-2080. Email: tamori@med.kobe-u.ac.jp

Number of Tables: 2

Number of Figures:4

Supplementary Table S1. Characteristics of study participants by waist circumstance category

Variables	Waist circumference category					*P*
	WC 1 (*n* = 2192, 20.2%)	WC 2 (*n* = 2197, 20.2%)	WC 3 (*n* = 2300, 21.2%)	WC 4 (*n* = 2011, 18.5%)	WC 5 (*n* = 2152, 19.8%)	
Body weight (kg)	45.7 ± 5.0	51.6 ± 5.6	56.8 ± 6.5	61.8 ± 7.2	71.4 ± 10.2	<0.001
BMI (kg/m^2)	18.7 ± 1.6	20.7 ± 1.6	22.2 ± 1.7	23.8 ± 1.8	26.9 ± 3.0	<0.001
Waist circumference (cm)	69.1 ± 3.6	76.6 ± 1.6	81.7 ± 1.4	86.7 ± 1.5	96.0 ± 6.0	<0.001
SBP (mmHg)	120.8 ± 17.3	124.0 ± 16.8	128.0 ± 17.7	129.8 ± 17.5	132.8 ± 16.5	<0.001
DBP (mmHg)	72.1 ± 10.9	74.3 ± 10.5	76.8 ± 10.9	78.2 ± 11.0	79.8 ± 10.7	<0.001
Creatinine (mg/dL)	0.66 ± 0.13	0.69 ± 0.15	0.72 ± 0.31	0.75 ± 0.31	0.76 ± 0.23	<0.001
eGFR (mL min^{-1} 1.73 m^{-2})	72.6 ± 12.5	73.0 ± 12.9	72.5 ± 13.0	71.5 ± 13.4	72.6 ± 15.6	0.045
AST (IU/L)	22.6 ± 5.8	22.5 ± 7.9	22.6 ± 9.8	23.1 ± 8.7	25.7 ± 12.0	<0.001
ALT (IU/L)	16.3 ± 6.2	17.7 ± 10.3	19.0 ± 10.3	21.9 ± 13.1	27.1 ± 17.6	<0.001
γGTP (IU/L)	24.2 ± 21.2	29.5 ± 42.4	34.3 ± 47.3	37.5 ± 39.9	44.5 ± 46.7	<0.001
LDL-C (mmol/L)	3.34 ± 0.77	3.40 ± 0.81	3.39 ± 0.83	3.40 ± 0.86	3.31 ± 0.87	0.056
HDL-C (mmol/L)	2.09 ± 0.45	1.89 ± 0.45	1.77 ± 0.43	1.62 ± 0.40	1.51 ± 0.38	<0.001
TG (mmol/L)	0.90 ± 0.48	1.09 ± 0.63	1.25 ± 0.71	1.45 ± 1.57	1.61 ± 0.99	<0.001
Fasting plasma glucose (mmol/L)	5.1 ± 0.8	5.2 ± 0.8	5.5 ± 1.0	5.6 ± 1.2	5.9 ± 1.4	<0.001
HbA_{1c} (mmol/mol)	37.6 ± 4.2	38.1 ± 5.3	38.9 ± 6.4	39.9 ± 7.1	41.5 ± 8.1	<0.001
Number of comorbidities	0.75 ± 0.67	0.93 ± 0.72	1.15 ± 0.77	1.32 ± 0.78	1.60 ± 0.81	<0.001
Smoking rate, *n*, (%)	147 (6.7)	191 (8.7)	231 (10.0)	242 (12.0)	303 (14.1)	<0.001
Physical activity, *n*, (%)	1036 (47.3)	1052 (47.9)	1062 (46.2)	918 (45.7)	817 (38.0)	<0.001
Medication						
Hypoglycemic drugs, *n*, (%)	22 (1.0)	54 (2.5)	96 (4.2)	129 (6.4)	267 (12.4)	<0.001
Antihypertensive drugs, *n*, (%)	238 (10.9)	380 (17.3)	548 (23.8)	640 (31.8)	1023 (47.5)	<0.001
Lipid-lowering drugs, *n*, (%)	336 (15.3)	457 (20.8)	525 (22.8)	551 (27.4)	742 (34.5)	<0.001
Metabolic syndrome, *n*, (%)	0 (0)	0 (0)	0 (0)	376 (18.7)	1239 (57.6)	<0.001

Data are mean ± SD or *n* (%). All abbreviations are defined in the text. *P* values were calculated with the Jonckheere-Terpstra trend test or the Armitage trend test as appropriate.

WC1 indicates the smallest waist circumference group.

Supplementary Table S2. Characteristics of study participants by waist circumstance category by gender

(a) Male

Variables	Waist circumference category					*P*
	WC 1 (*n* = 785, 22.0%)	WC 2 (*n* = 653, 18.3%)	WC 3 (*n* = 734, 20.5%)	WC 4 (*n* = 743, 20.8%)	WC 5 (*n* = 661, 18.5%)	
Body weight (kg)	56.1 ± 5.5	62.5 ± 4.7	66.3 ± 4.8	71.4 ± 5.2	80.4 ± 8.7	<0.001
BMI (kg/m^2)	20.2 ± 1.9	22.3 ± 1.4	23.5 ± 1.6	25.1 ± 1.7	28.0 ± 2.9	<0.001
Waist circumference (cm)	74.5 ± 4.0	81.4 ± 1.1	85.4 ± 1.3	90.2 ± 1.6	99.1 ± 5.9	<0.001
SBP (mmHg)	125.0 ± 16.8	130.4 ± 18.4	130.4 ± 17.7	131.5 ± 16.5	135.0 ± 16.3	<0.001
DBP (mmHg)	76.8 ± 10.4	79.9 ± 11.4	80.2 ± 10.8	80.7 ± 10.9	82.5 ± 10.9	<0.001
Creatinine (mg/dL)	0.84 ± 0.15	0.88 ± 0.51	0.87 ± 0.23	0.90 ± 0.43	0.88 ± 0.24	<0.001
eGFR (mL min^{-1} 1.73 m^{-2})	73.3 ± 13.7	71.8 ± 13.9	71.2 ± 13.8	70.0 ± 13.6	70.6 ± 14.7	<0.001
AST (IU/L)	23.5 ± 11.1	24.0 ± 14.0	24.1 ± 10.0	24.5 ± 9.6	27.7 ± 13.5	<0.001
ALT (IU/L)	18.4 ± 8.9	21.1 ± 13.3	23.5 ± 12.9	26.0 ± 16.5	30.4 ± 17.8	<0.001
γGTP (IU/L)	38.8 ± 62.0	47.3 ± 66.7	50.8 ± 61.4	51.7 ± 57.3	56.2 ± 55.8	<0.001
LDL-C (mmol/L)	3.06 ± 0.79	3.18 ± 0.82	3.18 ± 0.82	3.16 ± 0.83	3.17 ± 0.82	0.077
HDL-C (mmol/L)	1.79 ± 0.46	1.64 ± 0.45	1.52 ± 0.38	1.44 ± 0.36	1.35 ± 0.32	<0.001
TG (mmol/L)	1.08 ± 0.72	1.31 ± 0.80	1.55 ± 1.12	1.76 ± 2.39	1.81 ± 1.14	<0.001
Fasting plasma glucose (mmol/L)	5.46 ± 1.00	5.68 ± 1.22	5.82 ± 1.15	5.86 ± 1.23	6.19 ± 1.62	<0.001
HbA_{1c} (mmol/mol)	38.1 ± 6.1	39.4 ± 8.8	40.0 ± 8.0	40.5 ± 7.1	42.6 ± 9.2	<0.001
Number of comorbidities	0.81 ± 0.75	1.18 ± 0.84	1.35 ± 0.79	1.50 ± 0.84	1.80 ± 0.77	<0.001
Smoking rate, *n*, (%)	177 (22.6)	132 (20.2)	162 (22.1)	176 (23.7)	137 (20.7)	0.485
Physical activity, *n*, (%)	395 (50.3)	338 (51.8)	387 (52.7)	299 (40.2)	237 (35.9)	<0.001
Medication						
Hypoglycemic drugs, *n*, (%)	36 (4.6)	44 (6.7)	55 (7.5)	76 (10.2)	104 (15.7)	<0.001
Antihypertensive drugs, *n*, (%)	126 (16.1)	191 (29.3)	256 (34.9)	333 (44.8)	395 (59.8)	<0.001
Lipid-lowering drugs, *n*, (%)	92 (11.7)	117 (17.9)	154 (21.0)	201 (27.1)	213 (32.2)	<0.001
Metabolic syndrome, *n*, (%)	0 (0)	0 (0)	227 (30.9)	410 (55.2)	459 (69.4)	<0.001

(b) Female

Variables	Waist circumference category					*P*
	WC 1 (*n* = 1532, 21.1%)	WC 2 (*n* = 1448, 19.9%)	WC 3 (*n* = 1393, 19.1%)	WC 4 (*n* = 1448, 19.9%)	WC 5 (*n* = 1455, 20.0%)	
Body weight (kg)	44.2 ± 4.2	48.5 ± 4.0	51.7 ± 4.2	55.4 ± 4.7	63.2 ± 7.8	<0.001
BMI (kg/m^2)	18.5 ± 1.6	20.1 ± 1.5	21.5 ± 1.7	22.9 ± 1.9	26.2 ± 3.2	<0.001
Waist circumference (cm)	67.9 ± 3.4	74.8 ± 1.4	79.4 ± 1.2	84.3 ± 1.6	93.9 ± 6.2	<0.001
SBP (mmHg)	120.1 ± 17.5	122.7 ± 16.8	125.2 ± 17.3	128.3 ± 16.8	131.0 ± 17.1	<0.001
DBP (mmHg)	71.2 ± 10.8	73.5 ± 10.7	73.9 ± 10.4	76.2 ± 10.2	77.2 ± 10.5	<0.001
Creatinine (mg/dL)	0.64 ± 0.10	0.64 ± 0.11	0.64 ± 0.10	0.64 ± 0.13	0.64 ± 0.12	0.071
eGFR (mL min^{-1} 1.73 m^{-2})	72.4 ± 11.9	72.7 ± 12.7	73.1 ± 12.5	72.9 ± 13.2	73.7 ± 15.8	0.072
AST (IU/L)	22.7 ± 5.5	22.1 ± 5.5	21.9 ± 5.8	22.1 ± 8.1	24.2 ± 10.7	0.024
ALT (IU/L)	16.3 ± 6.1	16.6 ± 6.9	17.6 ± 10.9	19.1 ± 10.5	23.8 ± 16.5	<0.001
γGTP (IU/L)	22.9 ± 18.8	25.1 ± 22.8	25.7 ± 24.8	27.7 ± 22.4	31.9 ± 27.3	<0.001
LDL-C (mmol/L)	3.38 ± 0.76	3.48 ± 0.77	3.49 ± 0.80	3.55 ± 0.84	3.49 ± 0.87	<0.001
HDL-C (mmol/L)	2.14 ± 0.45	1.99 ± 0.44	1.86 ± 0.41	1.77 ± 0.42	1.67 ± 0.37	<0.001
TG (mmol/L)	0.87 ± 0.44	1.02 ± 0.56	1.13 ± 0.57	1.28 ± 0.68	1.40 ± 0.71	<0.001
Fasting plasma glucose (mmol/L)	5.06 ± 0.75	5.16 ± 0.70	5.23 ± 0.71	5.42 ± 0.91	5.64 ± 1.34	<0.001
HbA_{1c} (mmol/mol)	37.5 ± 3.7	38.1 ± 4.9	38.4 ± 4.6	39.2 ± 5.7	40.7 ± 7.5	<0.001
Number of comorbidities	0.73 ± 0.67	0.90 ± 0.69	1.06 ± 0.73	1.20 ± 0.74	1.44 ± 0.80	<0.001
Smoking rate, *n*, (%)	78 (5.1)	62 (4.3)	49 (3.5)	69 (4.8)	72 (5.0)	0.469
Physical activity, *n*, (%)	730 (47.7)	673 (46.5)	627 (45.0)	625 (43.2)	574 (39.5)	<0.001
Medication						
Hypoglycemic drugs, *n*, (%)	11 (0.7)	17 (1.2)	35 (2.5)	57 (3.9)	133 (9.1)	<0.001
Antihypertensive drugs, *n*, (%)	155 (10.1)	222 (15.3)	264 (19.0)	351 (24.2)	536 (36.8)	<0.001
Lipid-lowering drugs, *n*, (%)	232 (15.1)	293 (20.2)	379 (27.2)	392 (27.1)	538 (37.0)	<0.001
Metabolic syndrome, *n*, (%)	0 (0)	0 (0)	0 (0)	0 (0)	519 (35.7)	<0.001

Data are mean ± SD or *n* (%). All abbreviations are defined in the text. *P* values were calculated with the Jonckheere-Terpstra trend test or the Armitage trend test as appropriate. WC1 indicates the smallest waist circumference group.

Supplementary Figure S1. Prevalence of diabetes, hypertension, and dyslipidemia according to a quintile of waist circumference

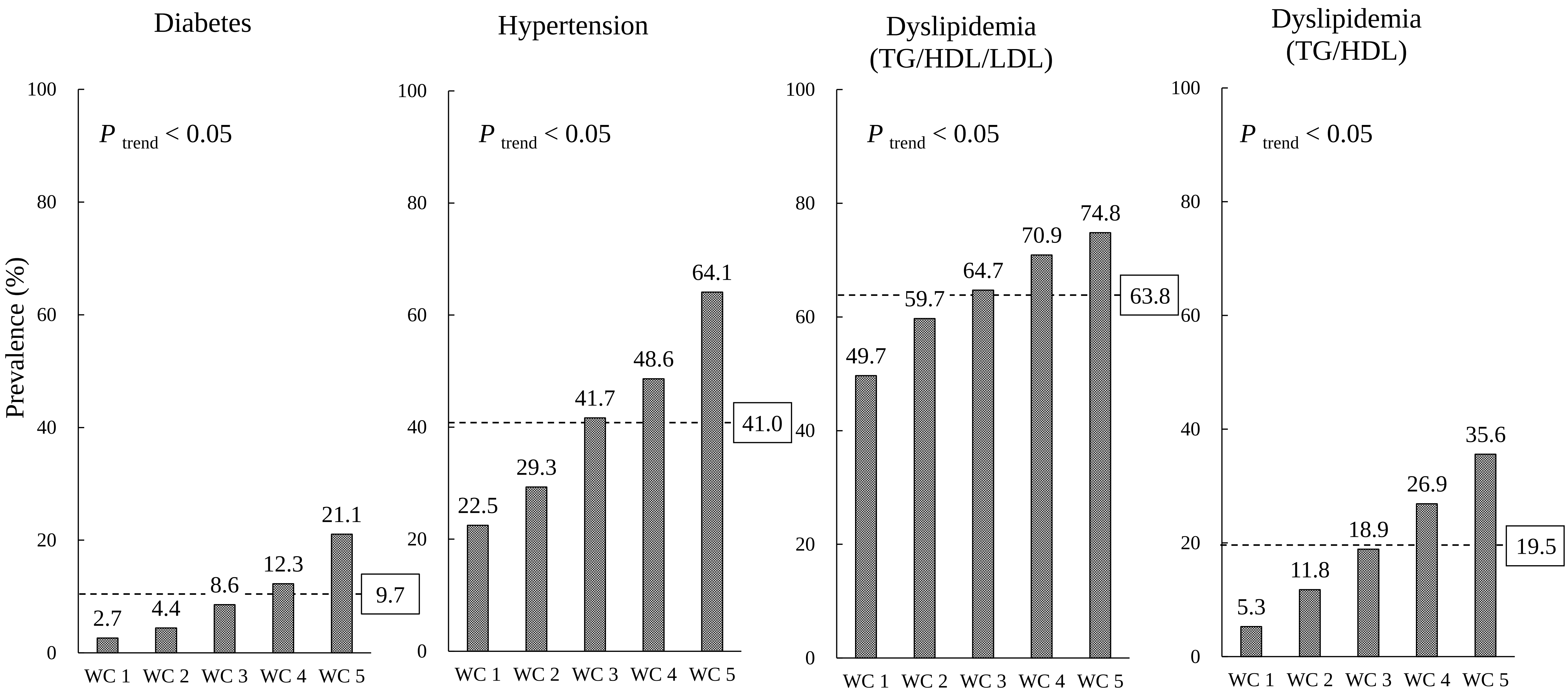

Numbers above bars represent prevalence (%). $P_{trend} < 0.05$ indicates a significant difference in disease prevalence among waist circumference categories as assessed by the Armitage trend test. The numbers in boxes indicate the prevalence of each disease among all participants. Dyslipidemia (TG/HDL/LDL) and dyslipidemia (TG/HDL) were defined in the text. WC1 indicates the smallest waist circumference group.

Supplementary Figure S2. Adjusted OR and its 95% CI for diabetes, hypertension, and dyslipidemia in participants of each waist circumference category of a quintile with the smallest circumference as the reference

Disease	WC category	OR	95%CI	P
Diabetes	WC 1	1		
	WC 2	1.51	[1.08,2.11]	0.059
	WC 3	2.80	[2.07,3.79]	<0.001
	WC 4	3.95	[2.93,5.32]	<0.001
	WC 5	7.11	[5.33,9.48]	<0.001
Hypertension	WC 1	1		
	WC 2	1.34	[1.17,1.54]	<0.001
	WC 3	2.18	[1.91,2.49]	<0.001
	WC 4	2.79	[2.44,3.20]	<0.001
	WC 5	5.05	[4.40,5.79]	<0.001
Dyslipidemia (TG/HDL/LDL)	WC 1	1		
	WC 2	1.61	[1.43,1.82]	<0.001
	WC 3	2.14	[1.89,2.42]	<0.001
	WC 4	2.98	[2.61,3.41]	<0.001
	WC 5	3.82	[3.34,4.38]	<0.001
Dyslipidemia (TG/HDL)	WC 1	1		
	WC 2	2.22	[1.76,2.79]	<0.001
	WC 3	3.63	[2.92,4.50]	<0.001
	WC 4	5.48	[4.42,6.79]	<0.001
	WC 5	7.81	[6.32,9.64]	<0.001

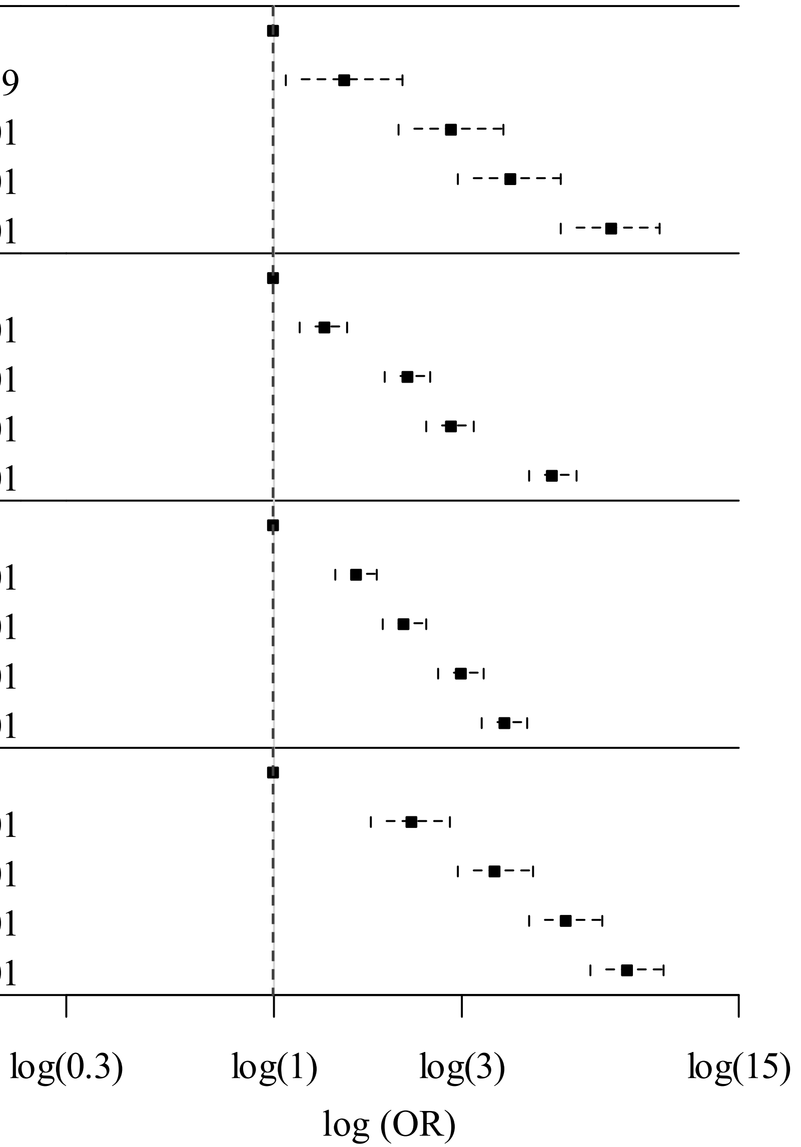

Each OR was adjusted for sex, smoking status, and exercise habit. The horizontal axis represents the log-transformed adjusted OR. P-values were calculated in the comparison of the OR for each disease between WC1 group (reference) and other groups by adjusting for multiplicity by Bonferroni's correction. Dyslipidemia (TG/HDL/LDL) and dyslipidemia (TG/HDL) were defined in the text. WC1 indicates the smallest waist circumference group.

Supplementary Figure S3. Prevalence of diabetes, hypertension, and dyslipidemia according to waist circumference classification by gender

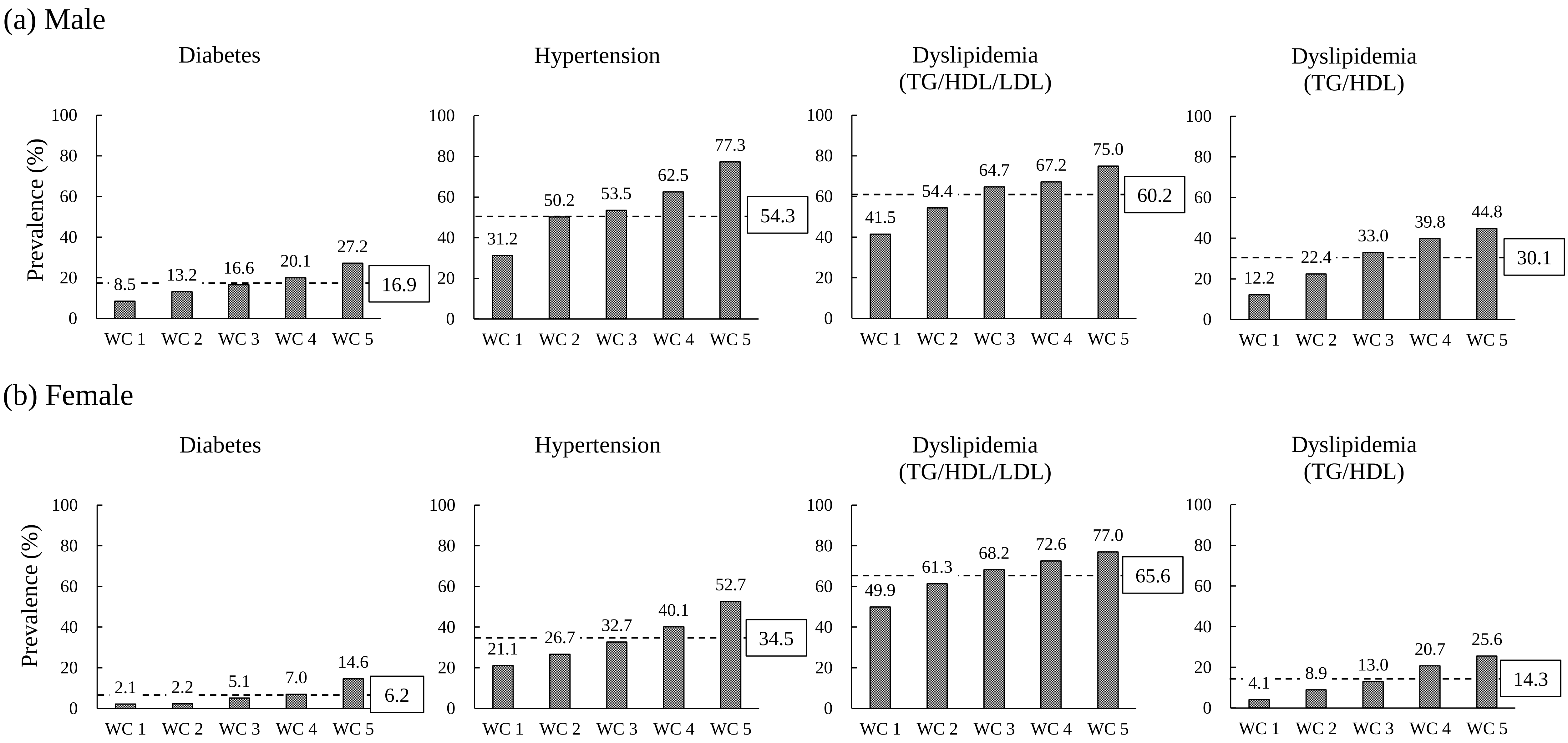

Numbers above bars represent prevalence (%). All analyses showed a significant difference in disease prevalence among waist circumference categories as assessed by the Armitage trend test. The numbers in boxes indicate the prevalence of each disease among each sexes of participants. Dyslipidemia (TG/HDL/LDL) and dyslipidemia (TG/HDL) were defined in the text.

Supplementary Figure S4. Adjusted OR and its 95% CI for diabetes, hypertension, and dyslipidemia in participants of each waist circumference category of a quintile with the smallest waist circumference (WC1) as the reference by gender

(a) Male

Disease	WC category	OR	95%CI	P
Diabetes	WC 1	1		
	WC 2	1.63	[1.16,2.28]	0.019
	WC 3	2.14	[1.56,2.93]	<0.001
	WC 4	2.69	[1.97,3.66]	<0.001
	WC 5	4.02	[2.96,5.45]	<0.001
Hypertension	WC 1	1		
	WC 2	2.23	[1.80,2.76]	<0.001
	WC 3	2.55	[2.07,3.14]	<0.001
	WC 4	3.63	[2.94,4.49]	<0.001
	WC 5	7.40	[5.84,9.38]	<0.001
Dyslipidemia	WC 1	1		
(TG/HDL/LDL)	WC 2	1.68	[1.36,2.07]	<0.001
	WC 3	2.58	[2.10,3.17]	<0.001
	WC 4	2.92	[2.37,3.60]	<0.001
	WC 5	4.33	[3.45,5.43]	<0.001
Dyslipidemia	WC 1	1		
(TG/HDL)	WC 2	2.13	[1.60,2.82]	<0.001
	WC 3	3.63	[2.78,4.73]	<0.001
	WC 4	4.76	[3.66,6.18]	<0.001
	WC 5	5.92	[4.54,7.72]	<0.001

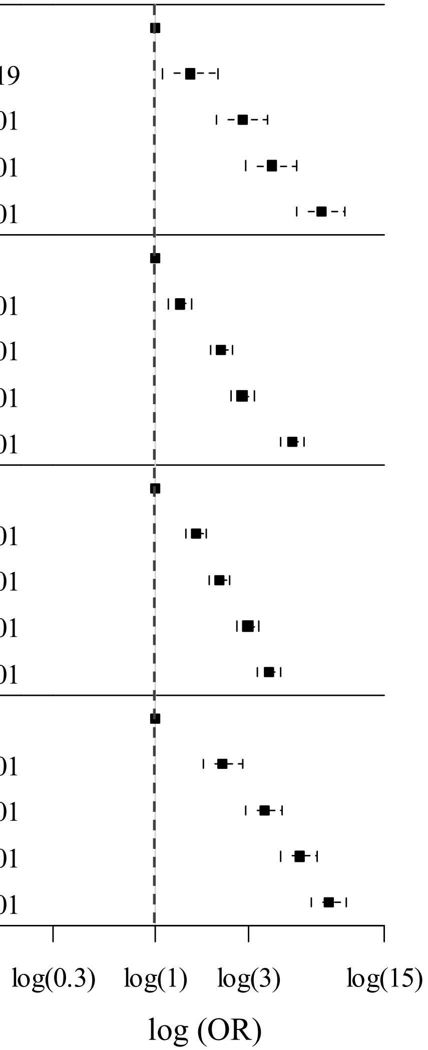

(b) Female

Disease	WC category	OR	95%CI	P
Diabetes	WC 1	1		
	WC 2	1.06	[0.65,1.74]	0.818
	WC 3	2.52	[1.65,3.85]	<0.001
	WC 4	3.51	[2.34,5.25]	<0.001
	WC 5	7.96	[5.44,11.63]	<0.001
Hypertension	WC 1	1		
	WC 2	1.36	[1.15,1.61]	0.002
	WC 3	1.81	[1.53,2.13]	<0.001
	WC 4	2.49	[2.12,2.93]	<0.001
	WC 5	4.12	[3.51,4.84]	<0.001
Dyslipidemia	WC 1	1		
(TG/HDL/LDL)	WC 2	1.59	[1.37,1.83]	<0.001
	WC 3	2.14	[1.84,2.49]	<0.001
	WC 4	2.64	[2.27,3.08]	<0.001
	WC 5	3.33	[2.84,3.90]	<0.001
Dyslipidemia	WC 1	1		
(TG/HDL)	WC 2	2.33	[1.71,3.19]	<0.001
	WC 3	3.58	[2.65,4.82]	<0.001
	WC 4	6.18	[4.65,8.21]	<0.001
	WC 5	8.13	[6.14,10.76]	<0.001

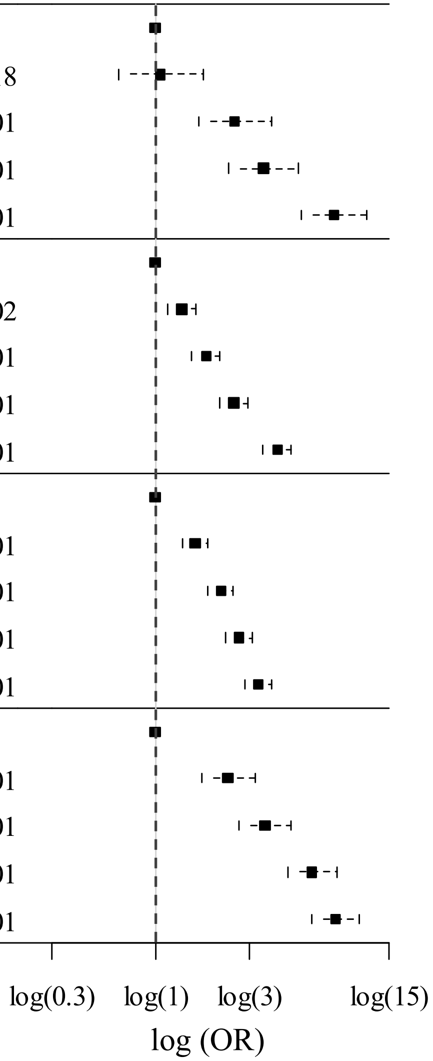

Each OR was adjusted for smoking status and exercise habit. The horizontal axis represents the log-transformed adjusted OR. P-values were calculated in the comparison of the OR for each disease between WC1 group (reference) and other groups by adjusting for multiplicity by Bonferroni's correction. Dyslipidemia (TG/HDL/LDL) and dyslipidemia (TG/HDL) were defined in the text. WC1 indicates the smallest waist circumference group. There was a significant intercategory difference in the OR between WC1 and other WC groups in all analyses except for diabetes between WC1 and WC2 groups in females.
